# Scaling up population health interventions from decision to sustainability – a window of opportunity? A qualitative view from policy-makers

**DOI:** 10.1186/s12961-020-00636-3

**Published:** 2020-10-09

**Authors:** Karen Lee, Femke van Nassau, Anne Grunseit, Kathleen Conte, Andrew Milat, Luke Wolfenden, Adrian Bauman

**Affiliations:** 1 School of Public Health, University of Sydney, Camperdown, NSW 2050 Australia; 2 The Australian Prevention Partnership Centre, Ultimo, NSW 2007 Australia; 3 Department of Public and Occupational Health, Amsterdam Public Health Research Institute, Amsterdam UMC, Vrije Universiteit Amsterdam, Amsterdam, The Netherlands; 4 Menzies Centre for Health Policy, School of Public Health and the University Centre for Rural Health, University of Sydney, Camperdown, NSW 2050 Australia; 5 Centre for Epidemiology and Evidence, New South Wales Ministry of Health, 100 Christie Street, St Leonards, NSW 2065 Australia; 6 University of Newcastle, Callaghan, NSW 2308 Australia

**Keywords:** Scale-up, Scalability, Sustainability, Decision-making, Policy-makers

## Abstract

**Background:**

While known efficacious preventive health interventions exist, the current capacity to scale up these interventions is limited. In recent years, much attention has focussed on developing frameworks and methods for scale-up yet, in practice, the pathway for scale-up is seldom linear and may be highly dependent on contextual circumstances. Few studies have examined the process of scaling up from decision to implementation nor examined the sustainability of scaled-up interventions. This study explores decision-makers’ perceptions from real-world scaled-up case studies to examine how scale-up decisions were made and describe enablers of successful scale-up and sustainability.

**Methods:**

This qualitative study included 29 interviews conducted with purposively sampled key Australian policy-makers, practitioners and researchers experienced in scale-up. Semi-structured interview questions obtained information regarding case studies of scaled-up interventions. The Framework Analysis method was used as the primary method of analysis of the interview data to inductively generate common and divergent themes within qualitative data across cases.

**Results:**

A total of 31 case studies of public health interventions were described by interview respondents based on their experiences. According to the interviewees’ perceptions, decisions to scale up commonly occurred either opportunistically, when funding became available, or when a deliberate decision was made and funding allocated. The latter scenario was more common when the intervention aligned with specific political or strategic goals. Decisions to scale up were driven by a variety of key actors such as politicians, senior policy-makers and practitioners in the health system. Drivers of a successful scale-up process included good governance, clear leadership, and adequate resourcing and expertise. Establishing accountability structures and appropriate engagement mechanisms to encourage the uptake of interventions were also key enablers. Sustainability was influenced by evidence of impact as well as good acceptability among the general or target population.

**Conclusions:**

Much like Kingdon’s Multiple Streams Theory of ‘policy windows’, there is a conceptually similar ‘window for scale-up’, driven by a complex interplay of factors such as political need, strategic context, funding and key actors. Researchers and policy-makers need to consider scalability from the outset and prepare for when the window for scale-up opens. Decision-makers need to provide longer term funding for scale-up to facilitate longer term sustainability and build on the resources already invested for the scale-up process.

## Background

The high economic and health costs of non-communicable diseases (NCDs), such as cancer and diabetes, is increasingly evident [1, 2], with NCDs accounting for nine out of ten deaths in Australia [3]. Consequently, Australian estimates are that $27 billion per year (36% of overall health expenditure) is spent on chronic diseases [3], much of which may be preventable by lifestyle modification in Australia alone [4]. A range of interventions to address lifestyle modification have been identified as effective when tested under research conditions and are recommended for implementation as part of national and/or global NCD action plans [5]. Despite this, the limited successful translation and scale-up of these interventions limit the evidence-base prevention benefit to the community [6].

‘Scale-up’ refers to the “*deliberate efforts to increase the impact of successfully tested health innovations to benefit more people and to foster policy and programme development on a lasting basis*” [7]. The concept of scale-up is not novel in public health. However, much of the experience of scaled-up interventions comes from communicable disease prevention in low- to middle-income countries, with limited evidence on scaling up NCD prevention efforts [6, 8]. In recent years, the proliferation of scale-up frameworks, theories, guidelines and models have contributed to understanding the nature of scale-up [9–12]. Scale-up requires assessment of the ‘scalability’ of interventions, developing a scale-up plan, garnering resources and implementing scale-up [10]. The ExpandNet model of WHO [7] suggests that scaling up generally occurs within specific environments (i.e. health needs, social and political contexts) with both ‘resource teams’ promoting or scaling up the intervention and ‘user organisations’ that adopt and deliver the intervention. Some frameworks imply that scale-up occurs in a linear fashion, with sequential steps following a considered decision-making process [13]. In practice, scale-up occurs through a variety of pathways [14] and is not always the result of deliberative policy decisions [15–17], but may be based on immediate policy priorities and individuals’ values, skills and experience [16, 17]. Finally, implicit in these scale-up frameworks is the assumption that scale-up follows proof of intervention effectiveness but decisions to scale-up are often made without sufficient evidence [16] and effective interventions can still take years to be translated into policy, if at all [18].

Few studies have examined the process of scaling up from the initial decision to implementation, particularly across different interventions and contexts [13]. The sustainability of scaled-up interventions is also infrequently investigated [19, 20] despite its importance to ensure that investments in scaled-up interventions are not wasted [21, 22]. The aim of this study was to obtain reflections from policy-makers on their experience of scaling up public health interventions within Australia, on the following research questions:

1. How were decisions to scale up made and what were the key influences for making those decisions?
2. Using reflections of the scale-up experience in the real world, what were the enablers of successful scale-up processes?
3. How many of the interventions that were scaled up were sustained and what influenced their sustainability?

## Methods

### Selection of interview respondents

We identified key Australian policy-makers, academics and practitioners with known experience in scaling up interventions in the area of health promotion and public health through professional networks of the study investigators. All potential respondents were contacted via email with an invitation to participate in the study. A snowballing technique was employed where initial respondents identified other relevant colleagues who were referred to the study. Respondents were eligible to participate if they had been directly involved in the scale-up of an intervention and/or played a role in the decision-making process for scaling up interventions.

### Interviews

Interview topic areas (Table 1) and questions were designed to obtain information regarding specific case studies of scaled-up interventions as well as broad reflections on the processes that policy-makers use to make decisions on scaling up health interventions. The interview schedule was semi-structured (Additional File 1) to allow the interview to be responsive to respondents’ answers and concepts emerging throughout the data collection process.

**Table 1:** Topic areas covered in interviews

Topic areas	Objective and description
Professional background	To obtain a description of the respondents’ professional background and experience on scaling up population health interventions
Participant’s experience in the scaling up of a population health intervention	To develop an understanding of a specific intervention(s) that had been scaled up, which included a description of the problem being addressed, the context in which it was scaled up as well as the process of scaling up
General reflection on the process of scaling up interventions, including outcomes of the scale-up process	To ascertain information pertaining to the key elements in the process of scaling up, such as identifying the key actors and their role, the role of evidence in decision-making and the key influences in the decision-making process as well as factors enabling or hindering potential sustainability

While there have been various attempts at defining what constitutes the ‘sustainability’ of health interventions [20, 23], to date, there has been no single agreed definition [24, 25]. For this reason, in this study, the study investigators have taken a pragmatic approach to defining sustainability, that is, interventions were categorised as being ‘sustained’ if they were successful in obtaining additional funding beyond the initial funding agreement and continued service delivery beyond the original scale-up period, which is consistent with previously used definitions of sustainability [20, 26] but is not as comprehensive as others have proposed [23, 25]. All interviews were conducted by one author (KL) with another (KC) co-interviewing in three of them, both of whom were female, degree-qualified and experienced qualitative interviewers and health researchers. Interviews were audio recorded and transcribed verbatim by an external transcription service (www.rev.com).

### Data analysis

The primary method of analysis of the interview data was the ‘Framework Analysis’ method [27, 28]. The ‘Framework Analysis’ is a qualitative method used to inductively generate common and divergent themes within qualitative data [28] and its defining feature is a matrix output of rows (generally cases, but in this study, interventions) and columns (themes/codes), where each ‘cell’ contains a summary of the qualitative data [27]. This method was well suited to this dataset as it enabled the examination of key themes across the entire dataset while allowing the views of each respondent to remain connected to the relevant intervention. The matrix of outputs was managed using Microsoft Excel. Applying an interpretive research approach to the data [29], the varying perspectives of respondents were used to construct a data-driven interpretation of how scale-up in public health programmes had occurred in the Australian context to date. Initial data analysis was conducted by the lead author (KL) and the interpretation modified and refined in discussion with other co-authors (KC, AG, AB, FvN). Final agreement on the key findings and results were reached in consultation with all authors.

### Ethics approval

Ethics approval for this study was obtained from the University of Sydney’s Human Research Ethics Committee Protocol No 2017/828. All participants gave written and verbal informed consent for the interviews to be recorded, transcribed and used for research purposes. All interview transcripts were de-identified for analysis and reporting. Quotes were de-identified to preserve the anonymity of the respondents and their affiliated organisations. All interview data during and after the study were stored in a secure, password-protected university server with access limited to the study investigators.

## Results

### Response rates and respondent characteristics

A total of 34 individuals were invited to participate in the interviews, of whom 29 agreed (response rate of 85%). The remaining invitees were not able to be contacted or did not respond to the initial invitation. Most interviews (*n* = 25) were conducted by telephone, with four as face-to-face interviews. Data collection was between December 2017 and September 2018 and the length of the interviews ranged from 25 to 82 mins with no repeat interviews conducted.

There was representation across respondents from all Australian States and Territories, although half were from the State of New South Wales. Organisationally, 21 were from state or regional health organisations, 4 from non-government organisations and the remaining 4 from academic institutions. The majority were policy-makers, with 44 at the senior level, 15 at the mid-level and 6 at the junior policy-maker level. In the Australian context, policy-makers are people employed by government organisations who set policy directions on behalf of political leaders. The remaining 4 held dual appointments as academics and clinicians. All respondents met the inclusion criterion of having participated in or led the scale-up of one or more health interventions; however, their level or depth of experience in scale-up overall was not obtained.

### Description of scale-up case studies

Respondents described 31 different case studies of public health interventions at varying stages of scale-up (Table 2). Some of the interventions had already been scaled up (*n* = 23), two were in process and four were still in the efficacy/effectiveness testing phase. A small number (*n* = 2) were intended for scale-up but ultimately did not progress. Almost all interventions were scaled up to the regional or state-wide level with one scaled up at the national level, although the exact numbers of sites the interventions were disseminated to within these levels was not collected, limiting any interpretations of the magnitude of scale-up across case studies.

**Table 2:** Overview of interventions

No	Intervention type	Setting	Scale-up outcome and/or status	Sustained
1	Lifestyle telephone counselling	General population	Scaled up	Yes
2	Diabetes prevention	Local health	Not scaled up	N/A
3	Childhood obesity programme	Local health	Currently in efficacy-testing stage	N/A
4	Mass media campaign for healthy lifestyle	General population	Scaled up	No
5	Culturally specific physical activity	Local health	Scaled up	Yes
6	HIV prevention	Local health	Scaled up	Outcome pending
7	Hepatitis C intervention	Non-health setting	Scaled up	Yes
8	Screening for hepatitis C	Non-health setting	Currently in efficacy-testing stage	N/A
9	Nutrition programme for pre-schoolers	Non-health setting	Adapted from original pilot and scaled up state-wide	Yes
10	Sugar-sweetened beverage reduction in children	Local health	Currently in efficacy testing stage	N/A
11	Childhood obesity prevention programme	Multiple settings	Scaled up	Yes
12	Physical activity for children	Local health	Scaled up	Yes
13	Early childhood and maternal services	General population	Scaled up and modified/adapted to merge with another at scale intervention	Yes
14	Nutrition and physical activity programme for children	Non-health setting	Modified and adapted from an earlier pilot study and scaled up	Yes
15	Falls prevention in community	Local health	Scaled up	Yes
16	Workplace health promotion	Non-health setting	Scaled up	Yes
17	Physical activity for adolescents	Non-health setting	Currently in effectiveness testing	N/A
18	Hepatitis C screening and treatment	Local health	Scaled up	Yes
19	HIV testing	Local health	Scaled up	Yes
20	Alcohol policy in sports	Non-health setting	Scaled up	No
21	Health promotion	Multiple settings	Scaled up	No
22	Chronic disease prevention	Multiple settings	Scaled up	Outcome pending
23	Workplace health promotion	Non-health setting	Scaled up	Yes
24	Workplace health programme	Non-health setting	Scaled up	Yes
25	Active transport	Non-health setting	Scaled up	Information unavailable
26	Sun protection	Non-health setting	In process of scaling up	N/A
27	Health promotion	Non-health setting	Not scaled up, replicated	Information unavailable
28	Childhood nutrition	Non-health setting	In process of being delivered at scale	N/A
29	Community nutrition	Non-health setting	Scaled up	Yes
30	Health promotion	Non-health setting	Scaled up	No
31	Sedentary behaviour	Non-health setting	Scaled up	Outcome pending

Many respondents indicated that they contributed to the decision-making process by providing evidence and advice to inform the scale-up decision, but their main role was in executing the scale-up process after the decision was made. Those few (*n* = 4) who claimed a direct role in the scale-up decision-making process were at senior executive level within state or territory health departments.

The public health interventions ranged from healthy lifestyles/health promotion interventions (*n* = 13), nutrition and physical activity (*n* = 9), diabetes or chronic disease prevention (*n* = 4), and communicable disease treatment and prevention (*n* = 5), although a number overlapped in these content areas. Intervention settings included local health districts/regions (*n* = 9), non-health environments such as schools, workplaces and/or sport settings (*n* = 16), multiple settings (*n* = 3), or the general population (*n* = 3). A full list of all the interventions can be found in Table 2 below.

Below is an analysis of our respondents’ personal accounts of the scale-up experiences of these interventions to answer our three main research questions.

### Key influences for making scale-up decisions

#### How decisions to scale-up were made

In Australia, decisions to scale-up commonly occurred in one of two ways; the first was opportunistic, when funding became available and opened a window of opportunity for scaling up interventions more broadly according to need. The second was when a clear decision was made to invest a priori in a particular intervention and to scale it up.

##### Scenario 1: Decisions to scale up were not tied to specific interventions

This describes a scenario where the initial ‘decision’ was made to address a general problem where the scale-up of a relevant intervention was a likely outcome. These decisions emerged when a health issue was deemed a Government priority or strategic target. Such decisions were often time bound within election cycles and accompanied by funding commitments and policy-makers had discretion on where and how funding was to be spent. Two real-world examples of this kind of opportunistic scale-up of interventions described by our respondents were either (1) through the National Partnership Agreement on Preventive Health (NPAPH) in 2009 [30] and (2) following state-level priority of child obesity. The NPAPH resulted from growing concern regarding the increasing prevalence of lifestyle-related chronic diseases and received funding over a 5-year period; this Agreement was considered the largest single investment provided by the Australian government in chronic disease prevention [30]. No specific interventions were mandated through this agreement but interventions targeted chronic disease prevention. The opportunity afforded by NPAPH prompted many policy-makers to consider scaling up existing interventions. The second example was where a state priority targeted the reduction of childhood obesity within a 5-year timeframe [31]. In order to meet the target, the time-bound sense of urgency resulted in scale-up of childhood obesity strategies. In both examples, the funding appeared in a rapid and unanticipated manner. Respondents described varying levels of ‘preparedness’, which suggested that the more prepared policy-makers were, the more efficiently they were able to plan for and assemble the resources required for scale-up.

“*It* [NPAPH] *was the largest amount of money health promotion had ever seen. And many States and Territories didn’t know what to do with it. But because* [PERSON A] *had been involved in negotiating that agreement at an inter-jurisdictional level,* [they were] *well advanced in thinking about what this investment could do … So there’s very much an element of strategic leadership and being ready to jump on that opportunity with something like* [this].” (P103)

However, there were no consistent processes for selecting the optimal intervention and selection was based on opportunity and availability. The majority of respondents noted that the key factors considered in deciding which interventions would be scaled up often included ‘new’ interventions that decision-makers perceived as being designed for scale or existing interventions that had evidence of effectiveness and could be integrated into existing service delivery structures. Where no intervention was readily available, new and untested interventions were scaled up and delivered at the population level without prior evidence of efficacy testing.

“*We just went from really basic research, and so on, straight into* [a] *big national programme… We put a proposal together and we at that point, hadn't even talked to any of the* [service delivery organisations]*. In fact, we wanted to spend the money immediately, even if there was a three-year timeline from the* [department] *because we wanted to put the money out, before the new change of government frankly.*” (P213)

##### Scenario 2: Decision to scale up a specific intervention/innovation

The second commonly described scenario was an a priori decision to scale-up specific interventions and funding was provided specifically for that purpose. Among our case studies, interventions were targeted for scale-up when they aligned with specific policy or strategic goals, a common driver of scale-up decisions. For example, the Australian government’s commitment to the eradication of both HIV and hepatitis C led to the rapid scale-up of interventions addressing these conditions.

“*So, around 2012-13 the* [jurisdiction] *committed to the virtual elimination of* [condition] *by 2020...What became clear was that we weren't really having the impact that we thought we did… so the* [politician] *had committed us to starting on* [date]*… She announced it and we started … and so we didn't have a lot of time really. That included procuring the* [treatment]*, getting all of the partners together... We started, and we just went from there.*” (P205)

#### Key influencers for scale-up decisions – key actors

According to the majority of our respondents, a range of ‘key actors’ in the health system were said to have a large influence on scale-up decisions. These key actors were described as politicians, senior policy-makers and executives, academics and clinicians, and community and advocacy groups. Academics, clinicians, community members or advocacy groups were reported to have influenced decisions through providing evidence of programme effectiveness or through public advocacy for a particular programme while mid-level policy-makers were reported to influence the process through access to senior decision-makers and feeding information to them.

“*I think it was really down to the champion leadership of two people…So they’re both very much focused on evidence-based practice and moving health promotion into scalable initiatives. So they were really crucial in making the arguments up through the decision-makers in the* [department] *that we actually needed to invest a significant amount of money if we wanted to make a significant change at a population level, and that there was evidence from other jurisdictions that this kind of work was possible, but it just had, literally, never been done anywhere in Australia before*.” (P102)

“*The social aspect and people really like it... and they finish it* [the intervention]*. I wouldn't be surprised if a whole lot of people went knocking on the door of Parliament House in some areas, because they're pretty solidly behind the programme…. There* [are] *votes in this. That's a broader issue, but we can't ignore that*.” (P209)

However, the ultimate decisions were made by politicians or executive level policy-makers and the perceived personal interests of such individuals were considered a key factor in decisions to scale up, irrespective of other considerations. Importantly, position hierarchy was also reportedly critical, where, despite prevailing evidence and advice, those in higher positions of power ultimately made final decisions.

“*What came along with that was the political will to do that… where we had a new Health Minister, who, amongst all of the things that* [they] *had to do, somehow took this one issue.* [They] *just wanted to drive it to make it happen*.” (P212)

“*That's the reality in policy settings because health policy-makers aren't always in control of what they need to do. They get instructed from on top… if we talk about the* [current intervention] … *we were instructed to do that. … it wasn't something that we thought we should be doing and the Ministers and the bureaucrats here were trying to block it a number of times, but basically it was imposed upon us by the [Person A]… and of course we wanted to make sure that whatever we did was as useful as possible, but if we were going to sit down do a crystal ball thing and we had this amount of money and this much health promotion resources, where would we make the best bang for our buck in terms of* [current intervention]*? We wouldn't have put it in [current intervention], that sort of call.*” (P221)

Often, when the decision to scale up was propelled by a sense of urgency (for example, to meet a strategic goal) it follows that the scale-up therefore had to happen quickly, even if it preceded testing the programme for evidence of effectiveness.

“*They were already looking at those results… ready to roll it out to the state. It was very, very quick. I think they really decided to roll it out to the state before I finished the final analysis. That's what I mean by enablers. I think the political ...there was a big push for the government to do as much as possible about childhood obesity. So, they saw the trend and they saw that it was promising, and they said, ‘Yeah, that's enough for us, and we're going with it’*.” (P206)

“*I think, and it was probably a mistake, because there were a lot of issues around the processes …and I seem to recall that they attempted to roll it out state-wide immediately… because there was a need to have state-wide programmes at the time and that was a policy need. .. in terms of just checking processes, it might have been useful to maybe pilot .... But we didn't have time to… we did a bit of piloting, but we were told that we had to turn it on and that the* [Person A] *was going to appear and cut the ribbon on the day before, and that we had to be all ready to go. We had to have a website all ready to go, but the website fell over the next day. So, we didn't have a period in which to test it… because the ribbon cutting had to happen*…” (P209)

Many respondents indicated that, while the availability of evidence was sometimes considered, it often came down to personal views of the key decision-makers and/or even the ideologies of the government that played a central role in intervention scale-up.

“*So I think that I would ... and we do a lot of policy work across government on a range of health issues, and research is almost irrelevant in the very beginning of the policy process. And that's slightly hard, but I mean, you know, 60% of the policies that are rolled out are done from an ideological, electoral position, and they may or may not be based on evidence*.” (P220)

### Enablers to a successful scale-up process

Key reported enablers of scale-up included a governance structure with clear leadership, adequate resourcing, appropriate outsourcing of scale-up delivery, implementing accountability structures, and adequate and appropriate engagement and communication with stakeholders. Regarding leadership, some respondents reported that scale-up via a top-down approach appeared to yield greater acceptance at the levels of service delivery and practice. Implementing a governance structure that clearly defined the roles and responsibilities of all parties and stakeholders involved in a successful scale-up process was also an enabler. Most frequently, well-articulated governance structures provided oversight of the scale-up process and was an important communication mechanism between the community and the government. In a few respondent accounts, devolving responsibilities to specific groups with appropriate expertise resulted in the governance structure working more efficiently and allowing input from diverse stakeholders.

“*We have expert data group. So, we review our data constantly really. You can get everyone around the table, all of your stakeholders around the table including the community, and the community are at this table, in our data expert working group to interrogate the data and agree on what it's saying. I think that's very powerful.*” (P212)

Almost all respondents reported that adequate resourcing (human and financial) was essential for scale-up. Scale-up with dedicated and well-resourced teams was generally accompanied by more positive experiences and was reportedly more successful. Sometimes, specific personnel were employed to manage the scale-up process, managing standardisation of the interventions and programme fidelity. Similarly, the opportunity for a central scale-up team meant that individualised support could be offered to delivery sites and this was perceived as a positive influence from both the scale-up team and the service delivery perspective.

“*The implementation was managed centrally, by a team and a programme manager, which supported the roll out. There is also a central ‘information portal’ that sites can be used to access information, and this is managed centrally as well*.” (P210)

Another enabler reported was the appropriate outsourcing to external organisations to deliver the scaled-up intervention. These external third-party organisations (frequently private consultancies) were thought to have greater flexibility to implement change as they were not hindered by internal organisational policies. In our case studies, these external organisations were used most frequently where they had specific expertise in the delivery setting, for example, in schools or workplaces or where the intervention being scaled up was licensed or trademarked, which meant that only those with specific skillsets or authority were able to deliver the intervention at scale.

“*So, we had an external implementer. Although we developed the programme, we got someone else to work day-to-day with the* [delivery settings]*, to see how they were going, and develop their programme. So, we gave them support. We had feedback from those contractors. Then developing the programme for any service to access, through our area. Which, who we got on board, because we knew, through the pilot, we were able to see how* [services] *access support. You’re better off having someone who already deals with* [delivery setting] *… than a new space*.” (P217)

Many of the respondents noted that some type of accountability structure was useful particularly for engaging service delivery organisations as part of the scale-up process. Strategies including key performance indicators and targets in funding agreements provided motivation for service delivery organisations to scale-up interventions. Linking elements of the intervention to existing organisational or industry policies and/or standards served as an enabler to motivate service delivery organisations to implement the intervention to meet accreditation or industry standards. Mandating ongoing data collection as part of the contractual arrangements helped service delivery agencies to monitor their scale-up progress and performance. Appropriately incentivising the service delivery workforce as part of the scale-up process was also considered important.

“*The* [scaled up intervention] *align with school practices, but the other carrot for teachers is their teacher accreditation. The initial training that they attend and any professional development that they do through these little courses, they're all accredited. Whether it be the school champion or the other PE teacher involved, it can contribute towards their own personal accreditation as well*.” (P210)

Finally, adequate and appropriate engagement and communication with a diverse range of stakeholders throughout all aspects of the scale-up journey was a significant enabler of the scale-up process. Positive impacts from stakeholder engagement included co-design of the intervention to be delivered at scale and an understanding of the range of end-user needs and expectations.

### Influences of sustainability for interventions that were scaled-up

Across the 23 interventions that were scaled-up in our study, 15 were thought to be sustained, that is, they obtained funding beyond the initial funding term and were able to continue service delivery. Within these, four received short-term funding only and had since ceased service delivery or had been absorbed into another programme. Some (*n* = 11) received longer-term funding and were still operating at the time of the interviews. The remaining five programmes were still operating within their initial funding term and the sustainability was therefore not yet known. Finally, four interventions were not sustained, that is, they ceased operations either prematurely or after the initial funding period had concluded.

Factors contributing to sustained interventions at scale included regular monitoring and reporting of the scale-up process as well as having sufficient evidence to demonstrate its value. Both quantitative and qualitative evidence from delivery agencies and end-user programme participants was used to demonstrate the intervention’s value. Most respondents indicated that decisions regarding maintenance of programmes at scale were more influenced by key actors in the system than by evidence of impact or reach. However, demonstrated impact generated wider community interest and advocacy, which raised the profile of the intervention, leading to greater attention from key decision-makers. This sometimes led to further ongoing and sustained support.

“*How to describe this ... in the ... following the publication of that original study, there was a slowly evolving, like pressure, expectation, from the* [government]*, …and the original research study, which was done in partnership with* [organisation A] *and* [organisation B]*, it was pretty high profile. So, it was short listed for* [a state award]*, and everybody knew about it. Interestingly, …. across the country, actually even internationally, everybody said, “Oh... how’d you do that?” And actually, also in the community sector, because originally it was a pretty radical notion … and it was pretty high profile. And so, because of those two things, and the expectation to increase, and the high profile,* [organisation] *might have had some years of funding to enhance the workforce and then, that's more or less continued over time. Not ongoing funding, but sort of one-year, or two-year tranches of money to do something more*.” (P205)

“*And so, we had two really good programmes* [intervention A and intervention B]. [Intervention A] *had some good research behind it too that was actually published. And you know, if you're looking at things like which programme actually has more reach, I'd actually say* [intervention A] *did. I think if I had to be the one to make the decision, if we could only keep one, I think I would have chosen* [intervention A]*… just in looking at scalability and reach, … I guess the reality of where different kinds of decisions come in. And I think it was a lot of politics involved, and…well, they lobbied. So, the non-government sector came in and lobbied the Minister directly. And the Minister instructed our Executive to not cut it* [intervention B]*. Whereas the* [other] *sector* [intervention A] *did not lobby*.” (P222)

Conversely, funding constraints represented a significant barrier to the sustainability of scaled-up interventions. All the case studies described in this study only occurred as a result of fixed-term funding agreements and few were successful in obtaining additional fixed-term funding to continue delivering or supporting the intervention at scale beyond its original term.

“*No, no. The one-off funding thing is never going to be scalable or sustainable. So… you have to consider: if it was to go on, what would it cost, and is that money there in the long term?*” (P208)

“*The issue of the cycle of poverty we have with prevention is that we fund things short term and then wonder why we're not getting the outcomes we thought we should ... so the sustainability has to be part of it*”. (P207)

In one case, shifting of funding from a (short) fixed term to being funded through the organisational operational budget was considered the optimal strategy to facilitate sustainability and this only occurred if there was sufficient evidence of impact and support from key decision-makers.

“*It was about reach and outcomes and we see the value of it as part of the prevention infrastructure. Like the QuitLine is part of the prevention infrastructure,* [intervention name] *is another part of the prevention infrastructure that’s available to the citizens of* [jurisdiction]*. So, we thought that our investment, that the outcomes that we got was worthy of the investment and that, because of the flexibility of the service into the future, and it’s a very flexible service, that it would’ve been crazy to stop funding*.” (P104)

In another case, a health intervention delivered in a non-health setting successfully demonstrated its impact; therefore, the ownership and funding of the intervention was transferred from its original health implementer to the non-health setting organisation and consequently maintained. This shifting of ownership and management of the intervention to another ‘organisation’ did not appear to have additional funding provided and, while it only occurred in one case study, it is nevertheless a potentially important strategy to facilitate sustainability.

## Discussion

These case studies described the different influences on decisions to scale-up and sustain population and preventive health interventions across Australia. They demonstrate that there is no consistent process or framework that could explain how scale-up occurs in the real world. Rather, scale-up is dependent on a complex interplay of factors, including the current policy context and government priorities in which scale-up is occurring. From our interviews, it appears that decisions to scale-up – whether they are opportunistic and rapid, or planned and strategic – are the result of an often fortuitous ‘window of opportunity’ that enables scale-up to proceed. This is contrary to the widescale promotion of theoretical models [32] to explain scale-up and these models need to be contextualised in real-world policy contexts.

Sustainability was dependent on a range of factors, with funding emerging as the most critical determinant of whether an intervention had the potential to extend beyond initial scale-up efforts.

**Fig. 1:**
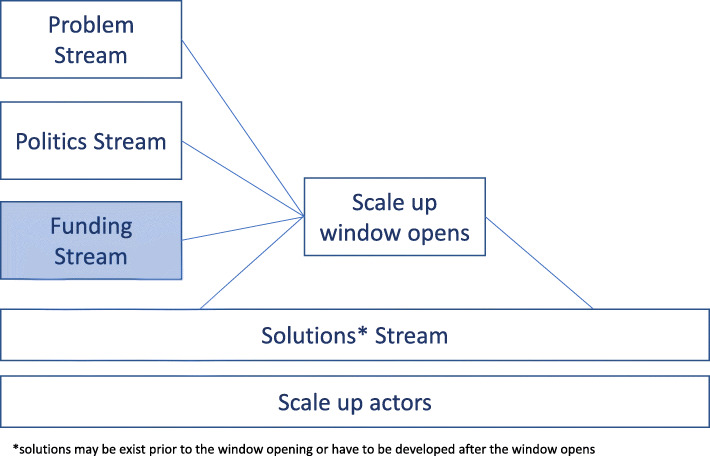
The ‘scale-up window’ - Adapted from Kingdon’s Multiple Streams Theory

The scale-up scenarios are analogous to Kingdon’s Policy Streams theory, where he posits that the problem, politics and solutions (i.e. interventions) streams need to converge in order for the policy window to open [33, 34]. In our study, a similar ‘window for scale-up’ became apparent, with the addition of a fourth stream that provides funding for scale-up; another difference is that scalable solutions may already exist prior to the window opening or may need to be developed after the window opens. The adapted schema for the ‘scale-up window’ process is illustrated in Fig. 1.

Underpinning these streams, is the role played by ‘scale-up actors’, analogous to Kingdon’s ‘policy entrepreneurs’ [17], who range from senior decision-makers to prominent community members, academics, clinicians or advocacy groups with the capacity to influence considerations of interventions for scale-up. Irrespective of whether an intervention gets scaled up opportunistically or by design, it is apparent that decisions are not driven by evidence nor research processes alone [16]. Rather, they are driven by factors such as political need, strategic context, funding availability and influence of key actors [13, 16, 34].

While it is not mandatory that all interventions that are scaled up must follow a rigorous research pathway, it is generally recommended [7, 10, 11, 16, 35, 36] so that resources are not wasted by scaling up sub-optimal or ineffective interventions. There is some evidence to suggest that the number of interventions being scaled up without prior efficacy or effectiveness is becoming more common [14], with mixed results, wherein some demonstrate effectiveness in evaluations post scale-up [37] and others demonstrating that the anticipated significant impact post scale-up was not achieved [38]. Further, programmes delivered at scale may produce smaller effects than observed in the original studies, a phenomenon known as the ‘voltage drop’ and this needs to be estimated or considered for each intervention delivered at scale [39]. Also evident is that these scale-up opportunities are usually unplanned and ad-hoc in nature. Until scale-up becomes a deliberately planned process, like most frameworks suggest it should be [7, 10, 11], policy-makers, practitioners and researchers advocating for scale-up will likely need to work within these fortuitous scale-up windows.

Previous studies have shown that the implications for policy-makers and researchers of such a context vary [16, 40]; policy-makers need to be ready to respond quickly to opportunities provided by new strategic priorities, budgeting processes or even during pre-election periods as they may provide openings to key decision-makers. They need to be agile and responsive and have a ‘drawer full of evidenced-based interventions’ that can be scalable and sustainable. Researchers, on the other hand, need to work collaboratively with policy-makers to develop interventions with scalability in mind from the outset, so that interventions can easily be adapted or integrated with existing services for scale-up [41, 42]. Co-creation between researchers and policy-makers/practitioners is an ongoing useful process to facilitate scale-up [43]. Rapid-response grant funding schemes should be available for rapid funding decisions to occur within the opportunistic scale-up windows [44, 45].

Arguably, a major limitation to scale-up is the unpredictable nature of policy and the personal values of decision-makers. Increasingly, other researchers have acknowledged the influence of power and politics and how key actors that hold that power may change the direction of health efforts [46, 47]. This can occur across a wide range of actors whose ideologies or personal values can influence how decisions are made at any level, ranging from high level policy decision-making to local level implementation. Our study confirms this influence and our respondents conveyed how the unpredictability and their inability to address this influence disrupted an evidence-based approach to scaling up. However, as Brownson et al. [40] indicated, despite the fact that policy-makers and researchers may have different priorities and seemingly different agendas, both need to work together to create opportunities to align evidenced-based interventions with existing priorities. They need to do so while engaging key and influential stakeholders and decision-makers throughout the whole process [42].

While it is likely that no scale-up process is the same, our case studies demonstrated that governance, resources, accountability mechanisms, workforce incentives, and engagement and communication consistently influence the scale-up process [8, 37, 48]. Engagement with stakeholders was undertaken at different stages but seemed to work better when co-design occurred early [42]. Similarly, our finding is that effective well-defined governance structures and scale-up resources are important to effective scale-up [8, 13, 37] as is the use of external consultants or independent flexible agencies that exist outside the internal government department policies and processes [13]. Furthermore, strategies that build in accountability in a way that motivates the stakeholders to scale-up interventions were positively received, particularly if accompanied by opportunities to examine reasons for failure to reach targets or to celebrate their achievements.

Finally, we identified factors influencing the sustainability of interventions at scale [21]. Regrettably, as others have found [49], few interventions were sustained over the longer term. While there has been some empirical research into the likely factors influencing sustainability [50, 51], in our case studies, we noted that, while impact was influential, other factors such as political expediency or influence were more important. Similarly, our case studies identified that longer-term funding commitments at scale are required as interventions delivered across diverse contexts need time to reach a certain level of maturity [21]. In absence of this, opportunities for embedding a scaled-up intervention in existing services or transferring of ownership are potential strategies that could be canvassed to facilitate sustainability. Further, to garner greater commitment, decision-making tools can assist policy-makers in making robust decisions about scaling up grounded in evidence of likely impact and success [35, 52, 53]. Another strategy could be the inclusion of sustainability in funding proposals or commitment to automatic renewal of funding that is contingent on meeting outcomes or demonstrating effectiveness.

### Study limitations

We acknowledge a number of limitations in this study. Firstly, all interview participants were based in Australia, although there was representation from every jurisdiction in Australia. A wider sample of participants from other countries, stakeholder groups and service delivery agencies may have provided different perspectives and is an area for further exploration. We also note that the experience of scale-up described here is limited to those described by our respondents and those of similar practitioners may differ. There was no follow-up or validation of the interview transcripts with the respondents. The results were reliant on personal accounts and retrospective recollections of processes as well as self-reported sustainability as narrated by the interview respondents. It is acknowledged that self-reported sustainability has been noted to be somewhat unreliable [24] and there was limited validation and/or triangulation of the intervention of events described, with minor exceptions. Despite this, personal descriptions are important as they reflect the real-world experience/accounts of the scale-up experience which is often not reported in any formal publications or reports. Secondly, this study did not specifically examine barriers in the scale-up process as this has been investigated previously by others [8, 37, 54]; however, this could be another area for further investigation in the future. To address some of these limitations, future prospective studies of scale-up are important and should be considered. Such studies, for example, could be operationalised through grant funding schemes that include specific scale-up criteria, against which the processes could be monitored prospectively. In addition, newly developed scalability tools [35, 42] could be used prospectively to identify and assess subsequent scale-up through tracking policy-makers from scale-up decision to actual scale-up. Finally, another area for future investigation could be a more in-depth examination of the role of the power held by key actors in shaping and steering decisions on scale-up.

## Conclusion

While multiple scaling up guides imply that scale-up might be a somewhat linear and logical process, our findings suggest that scale-up is often opportunistic and difficult to predict in real-world settings. When scale-up does occur, it is often accompanied by a sense of urgency to implement before the opportunity disappears. As such, scaling up an intervention may take precedence over considerations of the evidence and system readiness for scale-up. Within the climate of brief and unpredictable opportunities to implement interventions at a population level, researchers and policy-makers need to be agile and responsive to any opportunities. This can, to some extent, be achieved too by fostering closer ties between researchers and policy-makers to facilitate a bi-directional flow of knowledge resulting in making evidence-based interventions more feasible and appealing to policy-makers. In part, ensuring interventions are designed for scale-up would increase the likelihood that effective interventions are used at a population scale and sustained. Finally, long-term funding should be sought as a strategy to increase the sustainability of effective interventions at the population level as well as to increase opportunities to integrate scaled-up interventions into existing services or structures.

## Supplementary information

**Additional file 1.** Interview Guide.

## Data Availability

The datasets used and/or analysed during the current study are available from the corresponding author on reasonable request.
