# Supplementary material for: Scaling up population health interventions from decision to sustainability – a window of opportunity? A qualitative view from policy-makers
Source: Health Res Policy Syst. 2020 Oct 9;18:118. doi: 10.1186/s12961-020-00636-3 (PMC7547476; doi:10.1186/s12961-020-00636-3)
Supplement: Supplementary file 1 — Additional file 1. Interview Guide. [file 12961_2020_636_MOESM1_ESM.docx]

Additional File 1

Interview Guide

| **Topic: Professional background** | | |
| --- | --- | --- |
| **Interests** | **Possible questions** | **Objective** |
| Experience in scale-up | 1. Can you please describe your current role and your experience in scaling up interventions and the role you played in those? 2. What were some of the interventions that you have had experience in scaling up and what role did you play in those? | Informant’s perspective and experience |
| **Topic:** **Participant’s experience in a decision making process to scale-up a population health intervention**  *Ask the participant to* ***select a particular intervention*** *to discuss in detail in this section of the interview.* | | |
| **Interests** | **Possible questions** | **Objective** |
| Context | 1. Now, just wanting to unpack the process of scaling up a little more, from your experience, is there one intervention/program that has been scaled up in particular that you could describe the process in more detail? 2. Could we start by, firstly, describing the intervention and identifying what the need was for that intervention within the broader context? 3. So, why then was the intervention considered for scale-up? Prompts:  - resources available - political climate - evidence - alignment with strategic priorities | Describe background information and context for scale-up |
| Processes | 1. Can you describe what processes were undertaken in order to make the decision to scale-up the intervention? Prompts:  - Who was involved in these processes? - What was your role in these processes? | Describe the process of decision making |
| **Topic: Reflection on the process of scaling up interventions and sustainability** | | |
| **Interests** | **Possible questions** | **Objective** |
| Role of stakeholders | 1. I would now like just to get some general reflections on the process of scaling up interventions… In your experience, what roles do key stakeholders play in the process of scaling up population health interventions? | Understand the key stakeholders and their roles in scaling up |
| Identification and selection of interventions to scale-up | 1. In your experience, how are interventions for scale-up identified? 2. When multiple interventions are identified:  - How is one selected? - What is the selection process like? - What are the relevant considerations? | Examine how interventions are selected and compared |
| Role of Evidence | 1. In your experience, what is the role of evidence in the decision-making process?  - What kinds of evidence are used? (e.g. research evidence, expert opinion or other types of evidence) - How is evidence used? - What decisions does it inform? - What evidence is generally lacking that could inform a decision? | Explore the role of evidence in decision making |
| Influences on decision making | 1. In your experience, what are the most powerful influences on decisions to scale-up population health interventions? 2. What should they be? 3. If what is differs to what should be:  - Why is this so? - What could be done to correct it? | Examine other influences; compare and contrast the ideal with the status quo |
| Intervention / Scale-up implementation | 1. Now just going back to the intervention we discussed before…once a decision was made to scale it up how was the intervention implemented? 2. What were some of the enablers and/or barriers encountered?  - Modification of aims and objectives - Target groups - Engagement of stakeholders - Intervention governance - Funding and resources - Implementation - Evaluation | Examine implementation of interventions |
| Influences on scaling up | 1. In your experience, what are the key barriers to scaling up interventions? 2. What, if anything, would ameliorate these barriers? 3. What are the key considerations when scaling up? 4. How does the broader policy context influence scaling up? 5. What kind of information would be most useful to you if considering scaling up? | Explore influences on executing scale-up efforts |
| Influences on sustainability | 1. Was the intervention sustained beyond its intial funding term? 2. If so, what were some of the reasons for further funding? 3. If no, what were some of the reasons for it not being sustained? 4. In your experience what were some of the key influences that either determined it sustainability (or not) | Explore influences on sustainability |
